# Modeling DTA by Combining Multiple-Instance Learning with a Private-Public Mechanism

**DOI:** 10.3390/ijms231911136

**Published:** 2022-09-22

**Authors:** Chunyu Wang, Yuanlong Chen, Lingling Zhao, Junjie Wang, Naifeng Wen

**Affiliations:** 1 Faculty of Computing, Harbin Institute of Technology, Harbin 150001, China; 2 Department of Medical Informatics, School of Biomedical Engineering and Informatics, Nanjing Medical University, Nanjing 211166, China; 3 School of Mechanical and Electrical Engineering, Dalian Minzu University, Dalian 116600, China

**Keywords:** drug–target binding affinity, multi-instance learning, transformer

## Abstract

The prediction of the strengths of drug–target interactions, also called drug–target binding affinities (DTA), plays a fundamental role in facilitating drug discovery, where the goal is to find prospective drug candidates. With the increase in the number of drug–protein interactions, machine learning techniques, especially deep learning methods, have become applicable for drug–target interaction discovery because they significantly reduce the required experimental workload. In this paper, we present a spontaneous formulation of the DTA prediction problem as an instance of multi-instance learning. We address the problem in three stages, first organizing given drug and target sequences into instances via a private-public mechanism, then identifying the predicted scores of all instances in the same bag, and finally combining all the predicted scores as the output prediction. A comprehensive evaluation demonstrates that the proposed method outperforms other state-of-the-art methods on three benchmark datasets.

## 1. Introduction

Drug discovery aims to identify chemical compounds that can bind to targets involved in a certain disease. The identification of drug–target interactions (DTIs) plays a critical role in developing new drugs. The strength of the interaction between a drug–target pair can be determined by their binding affinity. Binding affinity is usually measured by biological experimental methods and expressed in measures such as the dissociation constant ($K_{d}$), the inhibition constant ($K_{i}$), or the half-maximum inhibitory concentration (IC50). Traditionally, an experimental assay is the surest way to obtain the desired binding affinity, but it is expensive and time-consuming to use this approach to analyze many possible DT pairs. A plethora of drug-like compounds and latent protein targets pose greater challenges because multiple drugs can be associated with multiple targets [1,2]. As a result, drug–target affinity (DTA) prediction has attracted considerable attention in recent years [3].

Existing works on DTA prediction can be categorized into (i) structure-based methods and (ii) structure-free methods. Structure-based methods rely on the 3D structure of the given target to explore potential binding sites. Molecular docking is a widely used structure-based approach for predicting the putative strengths of the proposed drug for binding to the target. In general, structure-based methods are more promising but cannot be employed if the tertiary structure of the protein of interest is unknown.

To overcome the current limitations of structure-based computational methods, a number of structure-free models have been developed for predicting DTA. In that context, deep learning (DL)-based DTA prediction approaches appear promising due to their ability to learn the underlying complicated mappings between the raw data of DT pairs and the corresponding affinity values. Such methods include DeepDTA [4], GraphDTA [5], DGraphDTA [6], WideDTA [7], GANsDTA [8], DeepCDA [9], ELECTRA-DTA [10], and DeepConv-DTI [11]. These methods employ different types of deep neural network techniques to process and extract contextual features from the input drug and target 1D or 2D information. However, previous works always followed a common paradigm, first extracting the drug and target global features with two separate deep neural encoders and then concatenating and subjecting the extracted global features to fully connected (FC) deep layers to predict DTAs. It can be observed that the resultant feature representations obtained before concatenation only present the respective properties of the drug or target, so they can be called “private features”; consequently, we call this paradigm the private late fusion mechanism, as the representations of drugs and targets are extracted by two independent encoders, and feature fusion occurs at the end of the model.

However, the private late fusion mechanism may lose the mutual information between drug–target (DT) pairs. DT pair interaction information should be memorized in the network to enhance the learning of pairwise occurrences and exploit the correlations available in the historical data. Following this route, various early fusion architectures have been proposed for DTA or DTI prediction tasks. The mutual learning (ML)-DTI method was developed with a cross-dependent design to allow the drug and target encoders to work together [12]. The graph-based early fusion affinity prediction (GEFA) method learns the joint representation of the input DT pair via an early fusion scheme [13]. The DeepAffinity method was developed with a joint attention model to fully explain the pairwise interactions between DT pairs [14]. The above studies have shown that both the private (separate) features and the public features (joint features obtained via early fusion) of DT pairs are closely related to the final DTA prediction results for the following reasons. In the entire DT space, DT pairs with interactions are sparse, making it difficult to learn effective low-dimensional public representations. Private encoding can capture the respective latent pattern information of drugs and targets without cross-interference; thus, private features may provide an important contribution to the final DTA score. Furthermore, public encoding explores feature interaction to learn the mutual patterns between drugs and targets in the joint representation space, which can reflect the DTAs from a different viewpoint. In total, both private and public feature representations extract key indicators from distinct perspectives and feature spaces for the DTA prediction task, and their complementarity can enhance the flexibility and efficiency of the resulting feature representation. Therefore, we construct a deep learning framework consisting of both private and public models for DT pair feature extraction.

As a variation of supervised learning, multiple instance learning (MIL) was first used to predict drug activity [15] and has since been applied to a variety of fields, including computer vision [16], medical imaging and diagnosis [17], and bioinformatics [18]. MIL is a method evolved from a supervised learning algorithm and defines a “bag” as a set of multiple instances for which a single class label is assigned. Actually, there are a variety of scenarios in which the classifications of objects (bags) can only be determined by some key components (instances), such as medical diagnoses; that is, some instances trigger the bag label. Following this concept, DTIs can be characterized by an MIL framework: the private representation contains abundant information that has been proven to be effective for DTA prediction [6,9,11,19], as does each public feature obtained via early fusion [2,12,13,14] and each public feature obtained via concatenation [4,5,7,20]. However, the exact contribution of each instance to the final DTA value of the bag is unknown. Therefore, we treat a DT pair as a bag; the private features, early fusion features and late fusion features extracted by deep neural networks are denoted as ‘instances’; and the binding affinity is considered the bag label. In this setting, each instance is used for the DTA prediction, and the deep neural network learns to capture the key instances and contributions of each instance.

In recent studies, deep MIL has achieved comparable performance to that of the state-of-the-art methods produced by combining different MIL approaches with a deep neural network model. Given that, we propose a deep MIL approach with a Private-Public Mechanism (called DMIL-PPDTA) to predict DTA. In summary, DMIL-PPDTA contains a sequence embedding mechanism, a multiple-instance generator and an MIL predictor. More specifically, sequence embedding is employed with transformer encoders to capture meaningful long-range relationships among the given sequences of drugs and targets. In the multiple-instance generator, the early fusion, late fusion and ligand-based methods for DTA are abstracted as bags by a private-public mechanism. Finally, the binding affinity is predicted by MIL regression based on these bags.

The rest of the paper is organized as follows: in Section 2, we report and analyze the performance of the proposed method. In Section 3 discuss the proposed method and the difference with others. In Section 4, we introduce the proposed method for predicting DT binding affinity. Finally, we conclude the paper in Section 5.

## 2. Results

In this part, we first describe the experimental settings, including the utilized datasets, performance evaluation metrics and baseline methods. Then, we compare our proposed DMIL-PPDTA method with the state-of-the-art models under the random dataset splitting and blind dataset splitting settings. Finally, we analyze the performance of DMIL-PPDTA in an ablation study to investigate the contribution of the proposed private-public mechanism.

### 2.1. Dataset

This study evaluated DMIL-PPDTA using three common benchmark datasets: the Davis dataset, the KIBA dataset, and the BindingDB dataset, as summarized in Table 1. Both the KIBA and Davis datasets comprise kinase proteins, while BindingDB contains more diverse protein families. The proteins in the Davis dataset vary more than those in the KIBA dataset; however, KIBA has a wider variety of compound types than the Davis dataset. The BindingDB dataset has much wider varieties of both drug and protein types than the Davis and KIBA datasets.

### 2.2. Evaluation Metrics

We used the following performance metrics to evaluate the DMIL-PPDTA model: the concordance index (CI), MSE, Pearson correlation coefficient (R), and $r_{m}^{2}$ index.

The CI metric measures whether the predicted binding affnity values are on the same order as their ground truths. It is computed as:

(1) $$\mathrm{CI}=\frac{1}{Z}\sum_{\delta_{x}>\delta_{y}}h\left(p_{x}-p_{y}\right)$$

where $p_{x}$ is the predicted value for a larger affinity $\delta_{x}$, $p_{y}$ is the predicted value for a smaller affinity $\delta_{y}$, *Z* is a normalization constant equal to the total number of samples, and h(m) is the step function [21]:

(2) $$h\left(m\right)=\left\{\begin{array}{cc} 1, & \mathrm{if}\;m>0,\\ 0.5, & \mathrm{if}\;m=0,\\ 0, & \mathrm{if}\;m<0. \end{array}\right.$$

The Pearson correlation coefficient (R) is a metric that measures the linear correlation between two variables. The Pearson correlation coefficient is calculated as in (3).

(3) $$R=\frac{cov(p,\delta )}{\sigma (p)\sigma (\delta )}$$

where cov indicates the covariance between the predicted value *p* and the real value δ, and σ represents the standard deviation.

The $r_{m}^{2}$ index is used to evaluate the external predictive potential of a model. $r_{m}^{2}$ is defined as:

(4) $$r_{m}^{2}=r^{2}*\left(1-\sqrt{r^{2}-r_{0}^{2}}\right)$$

where $r^{2}$ and $r_{0}^{2}$ represent the squared correlation coefficient values between the observed and predicted values with nonzero and zero intercepts, respectively. A model is acceptable if and only if $r_{m}^{2}\geq$ 0.5.

### 2.3. Baselines

We compared the proposed DMIL-PPDTA method with the previous state-of-the-art baselines: DeepDTA, GraphDTA, and ML-DTI. To maintain consistency with the state-of-the-art baselines, we followed the experimental parameter settings in their original publications.

**Figure 1:**
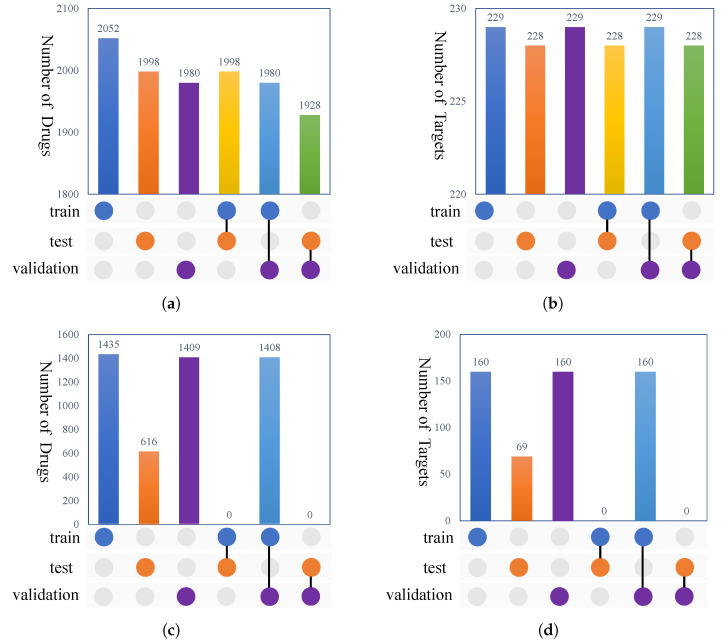
The overlap between drugs and targets in the KIBA dataset. (**a**) drug overlap under the random setting; (**b**) target overlap under the random setting; (**c**) drug overlap under the blind setting; (**d**) targets overlap under the blind setting.

Two experimental settings were used to evaluate the performance of our method. The first experimental setting was the random setting, where both the drug and target were randomly split for training and testing. In this case, the dataset was randomly divided into 5 folds, and 1-fold was chosen as the test set. We chose 80% of the remaining data as the training set and 20% as the validation set. Although the random setting is the most widely used splitting strategy to evaluate DTA models, it causes information leakage where the overlapping drugs and targets exist between the training and testing sets. In addition, in real world applications, one of the main challenges concerns the generalization abilities of DTA models. In other words, a DTA model should also predict the binding affinity for a new DT target pair. Therefore, we applied a blind setting where both the drugs and targets of the test set were unseen during training. In this case, the targets and targets were split at a 0.8/0.2 ratio for training–validation/testing. Then, the training set was split at a 0.75/0.25 ratio for training/validation. Figure 1 shows the overlaps between the targets and drugs under the random setting and blind setting for the KIBA dataset. From Figure 1, we can observe that no shared drugs and targets occurred between the training and testing sets under the blind setting.

### 2.4. Random Splitting

Table 2 compares the performance metrics of our DMIL-PPDTA model with those of the baselines on the Davis, KIBA, and BindingDB datasets under the random split setting. It can be observed that all four methods obtained $r_{m}^{2}$ values larger than 0.5 on these datasets, certifying the acceptability of the models. Among all the tested models, the proposed DMIL-PPDTA model performed best in terms of the average CI, MSE, R, and $r_{m}^{2}$ scores on the three datasets. On the Davis and KIBA datasets, all the models provided promising results, while DMIL-PPDTA obtained slightly better results than the baselines. The possible reason for this may lie in the fact that the DT pairs in the Davis and KIBA datasets are relatively dense; actually, the Davis dataset is a complete bipartite graph, and the sparsity of KIBA dataset is 24.9%, which causes the drugs or targets in the testing dataset to probably be seen during training.

The performance of all the tested models declined more or less on the BindingDB dataset. The DT pair distributions of BindingDB are sparse, which suggests that the predicted DT pairs are rarely seen during training, making the prediction process difficult. However, DMIL-PPDTA achieved a more significant improvement on this dataset than the baseline methods. DMIL-PPDTA obtained a 0.039 higher CI, a 0.246 lower MSE, a 0.065 higher R, and a 0.119 higher $r_{m}^{2}$ than the second-best scores achieved on the BindingDB dataset, indicating that the proposed DMIL-PPDTA model has better generalization ability.

Additionally, DeepDTA and ML-DTI provided close results on these datasets, while GraphDTA obtained slightly worse metric scores than those of DeepDTA and ML-DTI on the Davis and KIBA datasets.

### 2.5. Blind Setting

In an effort to provide a better assessment of our model, we conducted experiments with the blind setting to reveal the generalization abilities of DMIL-PPDTA and the baselines in a more realistic and challenging way. The blind setting means that the training and test sets did not share drugs and targets, ensuring that each protein–compound pair in the test set was unavailable in the training set.

Table 3 shows the results obtained under the blind setting. The performances of all four methods declined sharply from those obtained under random splitting. The $r_{m}^{2}$ values of the methods were much lower than 0.5, demonstrating that the generalization abilities of the models in the completely blind situations were limited and that improving the generalization ability of a DTI prediction model is still rather challenging. On the Davis dataset, these models’ performance degradations were most severe; specifically, their $r_{m}^{2}$ values were lower than 0.1, and their R values were lower than 0.3, indicating that the predictions deviated seriously from the ground truth. The reason for this could be that the blind setting made the training dataset divided from Davis too small, while deep learning-based methods tend to work well on large-scale datasets, thus resulting in the underfitting of all these models. In addition, the DMIL-PPDTA method obtained the best MSE but the worst CI, R and $r_{m}^{2}$ scores, although all of the models performed poorly, indicating that our model provided fewer average prediction errors than DeepDTA, GraphDTA and ML-DTI; however, its trend and order of the predicted binding affinities were more inaccurate than those of the other models. In contrast, the DMIL-PPDTA method obtained the best results in terms of all the metrics on both the KIBA and BindingDB datasets. This observation suggests that our model is more sensitive to the size of the utilized dataset due to its complex architecture and higher number of parameters. Furthermore, with the increase in the training dataset size (the training sets of the KIBA and BindingDB datasets are significantly larger than that of the Davis dataset), DMIL-PPDTA outperformed the baseline methods, certifying its better generalization ability even under this cold setting.

The results suggested that our molecular representation scheme could capture novel patterns on larger datasets for predicting the affinities of novel drug–target pairs better than the schemes of DeepDTA and ML-DTI, as the KIBA dataset is four times larger than the Davis dataset, and the BindingDB dataset is larger than the KIBA dataset. The reason for this might be that the early fusion scheme needs more data to extract hidden patterns under the blind setting than the late fusion method.

### 2.6. Ablation Study

In this section, we conducted an ablation study to evaluate the impact of each part of the private-public strategy on the BindingDB dataset. To show the contribution of each component, we decoupled each of them individually as follows:

1. Private instances only: In this case, only the private instance generator was utilized in DMIL-PPDTA.
2. Private and public-late instances: In this case, we removed the MHCA module from the public instance generator.
3. Private and public-early instances: In this case, we removed the concatenation scheme from the public instance generator.
4. Public-early instances only: In this case, only the MHCA module from the public instance generator was utilized in DMIL-PPDTA.
5. Public-late instances only: In this case, only the concatenation scheme from the public instance generator was utilized in DMIL-PPDTA.
6. Public-late and public-early instances: In this case, we removed the private instances from DMIL-PPDTA.

We report the comparison results obtained by the six versions of DMIL-PPDTA in Table 4; and it is not surprising that removing any part led to performance degradation. This confirms that all the modules in the private-public mechanism can learn implicit knowledge and enhance the prediction performance achieved in the DTA task. More specifically, both the model with the private and public-late instances and the model with the public-late and public-early instances achieved good performance, with CI values greater than 0.8. In particular, the model with private and public-late representations and the model with public-late and public-early representations achieved the second-best and third-best metrics (only inferior to those of the model with all instances), respectively, performing slightly worse than the model with private and public-late instances, indicating the strong contribution provided by public-late instances. However, each public or private instance alone did not provide sufficiently strong contributions.

## 3. Discussion

In this work, we extend computational methods in the field of drug discovery with multiple instance learning which is a popular variation of the supervised learning method. In addition, we employ the private-public mechanism with different fusion stages to capture interaction’s information better. The purpose of this study was to explore a different learning method and deliberate deep model for the DTA prediction problem with only raw sequence inputs. The existing works on DTA prediction mostly use different popular techniques to extract useful representations of drugs and proteins, and then the combined representation is fed into complex deep models to find the hidden complex relations between drugs and proteins. The representation learning part has been proven to be efficient in different methods. Some methods, such as DGraphDTA, DeepCDA, DeepConv-DTI and MolTrans, use private representation with late fusion, while some others, such as DeepDTA, GraphDTA, and WideDTA use public representation by early fusion. However, the contribution of either one to the model and the result is still unknown. Thus, we want to tackle this challenge more deeply and try to find some insights in the proposed DMIL-PPDTA method.

## 4. Materials and Methods

In this section, we formulate the DTA task as an MIL problem and present its deep learning model implementation.

### 4.1. Problem Formulation

In MIL, data are organized as labeled bags, each of which contains a number of instances. In the task of our DTA prediction model, each drug is represented by SMILES, and each target is represented by an amino acid sequence. A DT pair is regarded as a “bag” $X_{i}$ with its binding affinity as the label $y_{i}$. The private and public features generated by deep neural networks from the input DT pair are considered instances. Accordingly, we formulate a DT pair with multiple feature representations as $X_{i}=\left\{{\vec{x}}_{i,1},{\vec{x}}_{i,2},\ldots ,{\vec{x}}_{i,N}\right\}$, and the cardinality *N* is the number of instances. An instance ${\vec{x}}_{i,j}\in R^{d}$ lives in a *d*-dimensional feature space $R^{d}$. The binding affinity of the *i*-th DT pair is denoted as $Y_{i}$. The aim of our model is to identify the binding affinity of an unseen DT pair.

### 4.2. Model Architecture

**Figure 2:**
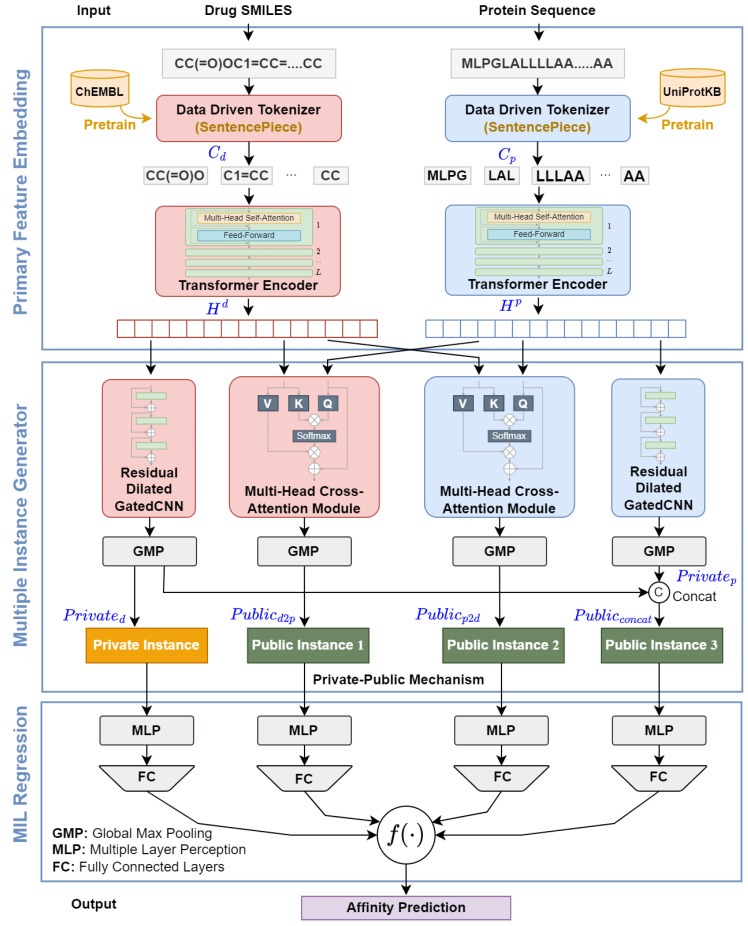
A graphical illustration of DMIL-PPDTA.

The proposed DMIL-PPDTA approach consists of three stages (shown in Figure 2): primary feature embedding, instance construction, and MIL pooling regression. In the primary feature embedding module, the amino acid sequence and SMILES are first tokenized using the SentencePiece algorithm and then embedded by two transformer encoders. In the instance construction module, private drug instances, early public instances, and late public instances are formed based on the primary feature embeddings provided by the private-public mechanism. On the top of the instance construction module, all instances are fed into FC layers to evaluate their binding affinity scores, and these scores are fused in the same bag as the binding affinity of the bag using linear regression.

### 4.3. Primary Feature Embedding

#### 4.3.1. Data-Driven Tokenization

Motivated by the domain knowledge that DTI produces at a substructural level, we employ a subword tokenization algorithm, namely, the SentencePiece Unigram algorithm, to segment the SMILES and amino acid sequences into tokens. The SentencePiece Unigram algorithm creates a vocabulary by modeling the probabilities of subwords that minimize the complexity of the language model. The multiresidue tokens that comprise the vocabulary subdivide low-entropy areas and reduce the overall length of the encoded sequences.

In our work, we pretrain two SentencePiece Unigram models to tokenize the amino acid sequences and SMILES; the protein tokenization model is trained on the 0.56 M protein sequences in UniProtKB [22], and the SMILES tokenization model is trained on the GuacaMol benchmark dataset [23] consisting of 1.6 M compounds curated from ChEMBL [24]. Herein, the DMIL-PPDTA approach converts the drug SMILES and protein sequences into sequences of substructures as $C_{d}$ and $C_{p}$, respectively, based on the pretrained SentencePiece Unigram models.

#### 4.3.2. Contextualized Embedding

To facilitate the learning of hidden patterns from the raw data, we use transformer encoders to enrich the embeddings based on the inputs $C_{d}$ and $C_{p}$. The transformer encoders encode $C_{d}$ and $C_{p}$ to learn contextualized drug and protein representations, respectively. A transformer encoder consists of a stack of *L* layers, each with two sublayers: a multihead self-attention layer and a feedforward layer.

Suppose we are given a sequence of embedding vectors $x\in R^{T\times h\times d}$, where *T* is the length of the sequence, *h* is the number of attention heads, and *d* is the dimensionality of each head. The *j*-th self-attention head projects the input x to a (query, key, value) triplet by learnable weights matrices $W_{j}^{q},W_{j}^{k},W_{j}^{v}\in R^{T\times d}$ as $\left(Q_{j},K_{j},V_{j}\right)$. Then, it computes the attention scores by performing the dot product operation between each pair of elements $K_{j}$ and $Q_{j}$. Utilizing the compared attention scores, the output of the *j*-th self-attention head is the weighted sum of $V_{j}$. Thus, the *j*-th self-attention head can be described as:

(5) $${\mathrm{head}}_{j}=\mathrm{softmax}\left(\frac{Q_{j}K_{j}^{⊤}}{\sqrt{d}}\right)V_{j},\;\;\;\;\;\;\;\;Q_{j}=xW_{j}^{q},K_{j}=xW_{j}^{k},V_{j}=xW_{j}^{v}.$$

The multihead self-attention mechanism is an extension of the single-head self-attention mechanism that can jointly model the multiple interactions from different representation spaces:

(6) $$\mathrm{MultiHead}\left(x\right)=\left[{\mathrm{head}}_{1};\ldots ;{\mathrm{head}}_{k}\right]W^{O}.$$

Next, a positionwise feedforward network (FFN) transforms the intermediate output of the multihead self-attention mechanism as follows:

(7) $$\begin{array}{c} \mathrm{FFN}\left(x\right)=\max\left(0,xW_{1}+b_{1}\right)W_{2}+b_{2}. \end{array}$$

Then, the above two components are connected with a residual connection and layer normalization [25]:

(8) $$\begin{array}{cc} \; & \mathrm{ResiNorm}(f,x)=\mathrm{LayerNorm}(x+f(x)),\\ \; & \mathrm{Encoder}(x)=\mathrm{ResiNorm}(\mathrm{FFN},\mathrm{ResiNorm}(\mathrm{MultiHead},(x))). \end{array}$$

In addition, since the self-attention mechanism ignores the order information of a sequence, a positional embedding PE is used to represent the positional information. Specifically, we employ a sinusoidal positional encoding scheme:

(9) $$\begin{array}{cc} {\mathrm{PE}}_{p,2i} & =\sin\left(p/10000^{2i/d_{\mathrm{model}}}\right),\\ {\mathrm{PE}}_{p,2i+1} & =\cos\left(p/10000^{2i/d_{\mathrm{model}}}\right), \end{array}$$

where *p* is the position and *i* is the dimensionality.

For a DT pair, the embedded DT representations are fed into two transformer encoders. Representation modules stack the *M* and *N* heads of the transformer encoders to encode the drug ($C_{d}$) and protein ($C_{p}$) embeddings, respectively. In particular, for drug $C_{d}$,

(10) $$\begin{array}{cc} H_{l}^{drug} & ={\mathrm{Encoder}}_{l}^{drug}\left(H_{l-1}^{drug}\right), \end{array}$$

(11) $$\begin{array}{cc} H_{1}^{drug} & ={\mathrm{Encoder}}_{1}^{drug}\left(\mathrm{Emb}\left(C_{d}\right)\right), \end{array}$$

and for protein $C_{p}$,

(12) $$\begin{array}{cc} H_{l}^{protein} & ={\mathrm{Encoder}}_{l}^{protein}\left(H_{l-1}^{protein}\right), \end{array}$$

(13) $$\begin{array}{cc} H_{1}^{protein} & ={\mathrm{Encoder}}_{1}^{protein}\left(\mathrm{Emb}\left(C_{p}\right)\right), \end{array}$$

where Emb represents the word and position embeddings and Encoder represents a transformer encoder layer. Formally, let matrix $H_{M}^{drug}\in R^{d\times n}$ be the output of the drug transformer encoder and $H_{N}^{protein}\in R^{p\times n}$ be the output of the protein representation module, where *d* is the length of a drug SMILES, *q* is the length of a protein sequence, and *n* is the dimensionality of the model.

### 4.4. Multiple-Instance Generator Based on a Private-Public Mechanism

In our implementation, we generate instances with a private-public mechanism. The private-public mechanism originates from various works on the DTA problem. The private part simulates ligand-based methods, and the public part integrates the early fusion and late fusion strategies. In this section, we give a detailed description of the formation of the multiple-instance generator including a private instance generators and a public instance generator.

#### 4.4.1. Private Instance Generator

**Figure 3:**
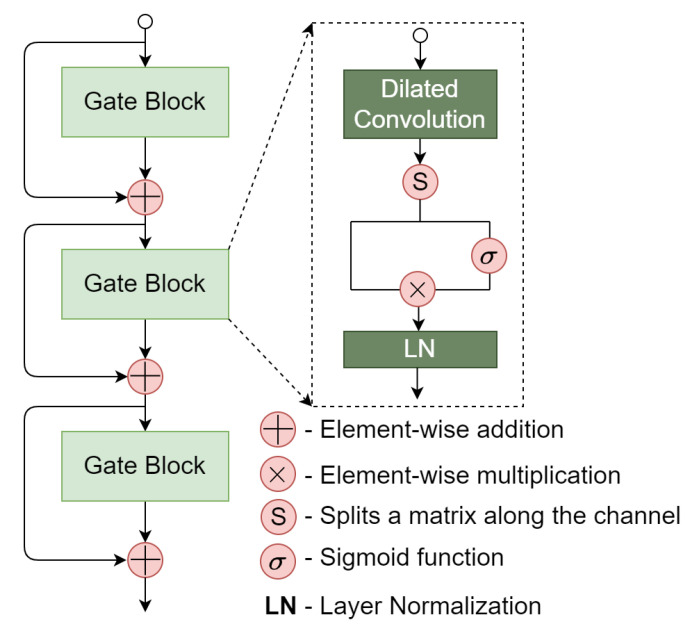
Illustration of the residual dilated GatedCNN module.

To further capture higher-level contextual information carried over by the protein sequence and drug SMILES, we propose a residual dilated gated convolutional neural network (GatedCNN) module based on the output of the transformer encoders. The residual dilated GatedCNN module is shown Figure 3. A main component of the residual dilated GatedCNN is the gate block. Given an input x, the gate block applies a 1D dilated convolution to capture higher-level contextual information. Different from the standard convolution, a dilated convolution can provide a long-range contextual field by skipping input values with a certain step *d*, which is otherwise known as the dilation rate. Specifically, the dilated 1D convolution operator $F_{d}\left(s\right)$ is defined as:

(14) $$F_{d}\left(s\right)=\left(x{*}_{d}f\right)\left(s\right)=\sum_{i=0}^{k-1}f\left(i\right)\cdot x_{s-d\cdot i}$$

where the filter f:{0,…,k−1}→R is a discrete function. *d* is the dilation rate, *s* is the index of the input element, and *k* is the filter size.

After a 1D dilated convolution layer, the output matrix *H* is divided (along the channel dimension) into two equal parts: $H\to [H^{l},H^{r}]$. Subsequently, $H^{r}$ is followed a sigmoid function that acts as a gate unit to choose the information of $H^{l}$ to be conveyed to the next layer. The operations can be formally described as follows:

(15) $$H^{o}=H^{l}⊗\sigma \left(H^{r}\right)$$

where σ is the sigmoid function and ⊗ represents elementwise multiplication. After performing layer normalization, the *i*-th gate block produces a matrix ${\hat{H}}_{i}^{o}$.

To mitigate the vanishing/exploding gradient problems, we employ the residual connection strategy in which the input of the i+1-th gate block is the sum of $H_{i}^{o}$ and the input of the *i*-th gate block:

(16) $$H_{i+1}^{o}={\hat{H}}_{i}^{o}+x_{i}.$$

Finally, a global max pooling (GMP) layer is used on the output of the residual dilated GatedCNN layers to avoid the overfitting problem and reduce the number of parameters. After completing the private feature extraction procedure, the high-level contextual features for proteins and drugs can be represented as ${\mathrm{Private}}_{P}\in R^{1\times m}$ and ${\mathrm{Private}}_{D}\in R^{d\times m}$, respectively. To simulate ligand-based methods, we only use the drug feature ${\mathrm{Private}}_{D}$ as a private instance.

#### 4.4.2. Public Instance Generator

**Figure 4:**
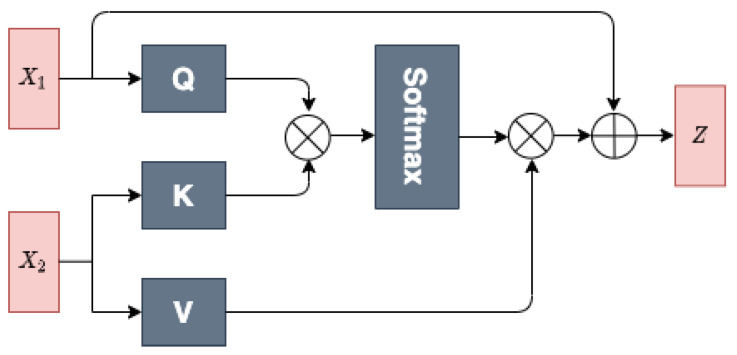
Multi-head cross-attention module.

DTI is a complex process involving biology and chemistry knowledge [26]. It has been proven that the interactive information between drugs and targets also plays a pivotal role in DTI prediction tasks [27]. In our method, the public instance generator focuses on modeling different levels of interaction information in high-dimensional spaces based on the drug and target features. More specifically, our public instance generator can generate two different levels of interaction features as public instances via a multihead cross-attention (MHCA) module (shown in Figure 4) and a concatenation scheme.

##### MHCA Module

The MHCA module is designed for extracting the interactive features between drugs and targets. The attention computes the query and key–value pair for obtaining the attention component, which is given by

(17) $$\mathrm{Attention}\left(Q,K,V\right)=\mathrm{softmax}\left(\frac{QK^{⊤}}{\sqrt{d_{k}}}\right)V$$

where **Q**, **K**, and **V** denote the query, key, and value, respectively. $d_{k}$ is the dimensional of the query. Intuitively, the multiplication operation between **Q** and **K** emphasizes the regions that slowly vary in time and have high power.

To exploit the drug–target interaction representations from different feature subspaces, MHCA is further employed to perform multiple attention function in parallel *h* times to generate queries, keys, values matrices $Q_{i},K_{i},V_{i}$ from i=1,…,h. Then, the outputs of independent attention are concatenated as the input of a linear transformation to obtain the interaction features, as shown in (18):

(18) $$\begin{array}{cc} \mathrm{MultiHead}(Q,K,V) & =\mathrm{Concat}\left({\mathrm{head}}_{1},\ldots ,{\mathrm{head}}_{h}\right)W^{O}\\ \mathrm{where}\;{\mathrm{head}}_{i} & =\mathrm{Attention}(QW_{i}^{Q},KW_{i}^{K},VW_{i}^{V}) \end{array}$$

where $W_{i}^{Q},w_{i}^{K},W_{i}^{V}$ are the weight matrices in parallel attention, and $W^{O}$ is the output weight parameter matrix. Taking $x_{1},x_{2}$ as input examples, queries are generated by $x_{1}$, and keys and values are produced based on $x_{2}$. Moreover, the output of the MHCA block **Z** is computed by:

(19) $$Z=X_{1}+\mathrm{MultiHead}\left(Q,K,V\right)$$

In this work, the MHCA module takes the output of the transformer embedding for a pair of drug targets, $D=H_{M}^{drug}\in R^{d\times n},P=H_{N}^{protein}\in R^{p\times n}$, as inputs. We compute public feature ${\mathrm{Public}}_{drug\to protein}$ by inputting $H_{M}^{drug}$ as *Q*, $H_{N}^{protein}$ as *K* and *V*, and we compute public feature ${\mathrm{Public}}_{protein\to drug}$ by inputting $H_{N}^{protein}$ as *Q*, $H_{M}^{drug}$ as *K* and *V*. With this special input method, the MHCA module determines the most relevant protein part for the drug and the most relevant drug part for the protein. Finally, GMP is also applied on the public features ${\mathrm{Public}}_{drug\to protein}$ and ${\mathrm{Public}}_{protein\to drug}$ to obtain two public instances.

##### Concatenation Scheme

In addition, we adopt a simple concatenation scheme based on a private instance generator to reflect the late fusion DTI information. We denote this public instance as:

(20) $${\mathrm{Public}}_{\mathrm{concate}}=\left\{{\mathrm{Private}}_{D},{\mathrm{Private}}_{P}\right\}.$$

### 4.5. Binding Affinity Prediction with MIL

The binding affinity prediction problem with MIL arises when each bag (DT pair) is made of multiple instances (private and public instances) corresponding to the same real-valued label (binding affinity). More specifically, this problem is a regression task; as opposed to classification, one cannot simply use the max function to identify positive instances as in the ordinary MIL method. Instead, we need to estimate the contributions of the instances toward the bag label. Therefore, we take the weighted linear combination of the instances as the final binding affinity value as (21), where the weights $w_{1},w_{2},w_{3}$ and $w_{4}$ are automatically learned during training:

(21) $$\hat{y}=w_{1}*{\mathrm{Public}}_{drug\to protein}+w_{2}*{\mathrm{Public}}_{protein\to drug}+w_{3}*{\mathrm{Public}}_{\mathrm{concate}}+w_{4}*{\mathrm{Private}}_{D}$$

Since the essence of DTA prediction is a regression task, we use the mean squared error (MSE) as the loss function. Let $\hat{y},y$ represent the predicted and real binding affinities, respectively, and let *N* be the number of samples. The MSE can be formulated as:

(22) $$\mathrm{MSE}=\sum_{i=1}^{N}{\left(y_{i}-{\hat{y}}_{i}\right)}^{2}.$$

### 4.6. Implementation Details

The implementation of each part of the proposed method is detailed below. The maximum lengths of the substructures of the protein sequence and SMILES were set to 512 and 128, respectively. The maximum lengths of the protein sequence and SMILES were set to 1024 and 256, respectively, from the atom-level view. We set the embedding dimensionality as 128 for all inputs. The vocabulary size for the drug substructures was 900, and the vocabulary size for the protein substructures was 10,000. As a result, we constructed context matrices with $M_{S}^{D}\in R^{128\times 128},M_{S}^{T}\in R^{128\times 512},M_{C}^{D}\in R^{128\times 256},M_{C}^{T}\in R^{128\times 1024}$. For the transformer encoder blocks, we set the number of layers to 2 and the number of multihead attention heads to 4. For the GatedCNN blocks, we set the kernel size to 3 for all CNN layers and the number of filters to 128,128,128. A multilayer perceptron (MLP) layer containing four FC layers with 1024,1024,512,1 neuron nodes was utilized to predict the binding affinity values. The MLP layers were also utilized as the feature extractor for the representations of drugs and proteins. The proposed framework was implemented using PyTorch 1.7. The model was optimized by AdamW with a learning rate of $1\times 10^{-3}$, betas of (0.9,0.999), and an Eps of $1\times 10^{-8}$. An early stopping technique specified the number of training epochs. Our experiments were run on Linux 16.04.10 with an Intel(R) Xeon(R) E5-2678 v3 CPU @2.50 GHz and a GeForce GTX 1080Ti GPU (11 GB).

## 5. Conclusions

In this study, a computational method for the DTA task is modeled using deep MIL for the first time. This method, DMIL-PPDTA, includes data-driven tokenization, an instance generator and MIL regression. More specifically, massive unlabeled drug SMILES and protein sequences are utilized to construct the data-driven tokenizer. Then, the original DT pair sequences are tokenized, and private and public instances are formed by the deep learning model. Accordingly, the DTA problem is formulated as a multiple-instance regression task for more effective prediction.

To evaluate the proposed method, the DMIL-PPDTA method was applied to the Davis, KIBA, and BindingDB datasets. The performance of the proposed DMIL-PPDTA model significantly surpassed that of ML-DTI, DeepDTA, and GraphDTA under the random splitting setting. Although the novel DTA prediction task under the blind setting was still challenging and extensive studies are still needed to improve the generalization abilities of the tested models, the DMIL-PPDTA model achieved competitive results, especially on the BindingDB dataset. We also conducted ablation experiments for the DMIL-PPDTA model under the random splitting setting on the BindingDB dataset. The results checked the importance of the instances to the final DTA prediction results and confirmed that the model containing all of the instances performed best.

Thus, we hope that this work will help researchers choose and devise new models that can achieve improved DTI prediction performance. As suggested by numerous studies, the enrichment of DT pair representations possibly improves the performance of DTA predictors, and we will explore the topological graph-based representations of DT pairs.

Although DMIL-PPDTA demonstrates good performances, there is still room for further improvements. (1) From the experimental results, all the models show different degrees of performance degradation under blinding setting. This is a kind of classical out-of-distribution (OOD) problem which means that neither the tested drugs nor targets appear in the training set. Thus, the more effective method should be designed to improve the generalization ability over the OOD test set. (2) We formulate the DTA problem as an MIL task, which actually is a Multiple Instance Regression problem. In this work, we utilized the linear regression to fuse the instances information, and the application-specific fusion method would be beneficial to the performance improvement of predicting drug–target binding affinity.

## Figures and Tables

**Table 1:** The detailed statistics of the datasets.

Dataset	# of Targets	# of Drugs	# of Interactions	Sparsity
Davis	361	68	24,548	1
KIBA	229	2052	117,184	0.249
BindingDB	1615	129,109	144,525	0.0007

# means total number.

**Table 2:** Comparison among the results obtained by the DMIL-PPDTA approach and the baseline methods across the datasets under the random splitting setting. The entries in boldface denote the best result for each metric, and the data in brackets represent standard deviations.

Dataset	Method	CI	MSE	R	$r_{m}^{2}$
Davis	DMIL-PPDTA	**0.880(0.007)**	**0.223(0.012)**	**0.810(0.011)**	**0.642(0.017)**
	DeepDTA	0.875(0.006)	0.239(0.019)	0.802(0.008)	0.571(0.026)
	GraphDTA	0.866(0.005)	0.240(0.009)	0.793(0.003)	0.621(0.009)
	ML-DTI	0.863(0.005)	0.234(0.012)	0.802(0.009)	0.601(0.032)
KIBA	DMIL-PPDTA	**0.881(0.003)**	**0.147(0.005)**	**0.888(0.003)**	**0.784(0.006)**
	DeepDTA	0.868(0.001)	0.188(0.002)	0.857(0.002)	0.697(0.014)
	GraphDTA	0.838(0.003)	0.208(0.005)	0.838(0.005)	0.696(0.012)
	ML-DTI	0.861(0.002)	0.189(0.003)	0.854(0.004)	0.702(0.015)
BindingDB	DMIL-PPDTA	**0.819(0.002)**	**0.754(0.013)**	**0.830(0.003)**	**0.685(0.008)**
	DeepDTA	0.778(0.005)	1.038(0.041)	0.762(0.009)	0.548(0.009)
	GraphDTA	–	–	–	–
	ML-DTI	0.780(0.007)	1.018(0.038)	0.765(0.011)	0.566(0.018)

**Table 3:** Comparison among the results produced by our DMIL-PPDTA approach and the baseline approaches across the datasets under the blind setting. The entries in boldface denote the best result for each metric, and the data in brackets represent standard deviations.

Dataset	Method	CI	MSE	R	$r_{m}^{2}$
Davis	DMIL-PPDTA	0.555(0.055)	**0.586(0.109)**	0.124(0.086)	0.022(0.016)
	DeepDTA	**0.630(0.036)**	0.771(0.236)	**0.270(0.054)**	**0.073(0.029)**
	GraphDTA	0.618(0.030)	0.787(0.077)	0.235(0.088)	0.061(0.047)
	ML-DTI	0.626(0.038)	0.725(0.146)	0.246(0.063)	0.062(0.028)
KIBA	DMIL-PPDTA	**0.655(0.009)**	**0.536(0.030)**	**0.488(0.022)**	**0.229(0.025)**
	DeepDTA	0.642(0.007)	0.591(0.046)	0.453(0.030)	0.182(0.024)
	GraphDTA	0.597(0.014)	0.633(0.031)	0.369(0.023)	0.125(0.016)
	ML-DTI	0.633(0.015)	0.614(0.014)	0.412(0.031)	0.147(0.022)
BindingDB	DMIL-PPDTA	**0.642(0.011)**	**2.020(0.053)**	**0.451(0.023)**	**0.188(0.021)**
	DeepDTA	0.618(0.007)	2.397(0.106)	0.383(0.021)	0.126(0.012)
	GraphDTA	–	–	–	–
	ML-DTI	0.620(0.011)	2.340(0.125)	0.391(0.025)	0.131(0.014)

**Table 4:** Ablation experiment results obtained by the DMIL-PPDTA approach on the BindingDB dataset under the random splitting setting. The entries in boldface denote the best result for each metric, and the data in brackets represent standard deviations.

Private	Public-Late	Public-Early	CI	MSE	R	$r_{m}^{2}$
✓			0.732(0.012)	1.357(0.081)	0.667(0.025)	0.418(0.024)
✓	✓		0.815(0.005)	0.779(0.038)	0.825(0.008)	0.664(0.029)
		✓	0.800(0.018)	0.881(0.113)	0.799(0.029)	0.623(0.047)
		✓	0.799(0.013)	0.889(0.092)	0.798(0.021)	0.609(0.052)
	✓		0.811(0.014)	0.807(0.103)	0.818(0.024)	0.655(0.056)
	✓	✓	0.815(0.010)	0.788(0.079)	0.823(0.016)	0.667(0.040)
✓	✓	✓	**0.819(0.002)**	**0.754(0.013)**	**0.830(0.003)**	**0.685(0.008)**

## Data Availability

Not applicable.
